# Honing in on magnetic resonance imaging predictors of multiple sclerosis pathology

**DOI:** 10.1111/bpa.13209

**Published:** 2023-08-30

**Authors:** Klaus Schmierer

**Affiliations:** ^1^ Faculty of Medicine & Dentistry The Blizard Institute, Centre for Neuroscience, Surgery & Trauma, Queen Mary University of London London UK; ^2^ Clinical Board Medicine (Neuroscience), The Royal London Hospital, Barts Health NHS Trust London UK

**Keywords:** multiple sclerosis, MRI/pathology correlation, myelin, post‐mortem, quantitative MRI, remyelination

As our understanding of the mechanisms leading to tissue damage and repair in multiple sclerosis (MS) evolves, so does our desire to detect and monitor their structural and functional correlates in people with this chronic disease during life. Given the heterogeneous nature of the tissue injury in MS, in terms of severity and timing, this is of significant interest for (i) the management of people with MS (pwMS) in clinical practice and (ii) the selection of outcomes in clinical trials.

However, very few biopsies are being undertaken to confirm a tissue diagnosis of MS or to assess specific lesion stages, and despite significant progress in the field of fluid biomarkers [1], magnetic resonance imaging (MRI) retains its pivotal role translating histological findings into MRI signals that can be measured repeatedly, over a virtually infinite number of time points.

The desire to directly correlate changes identified using MRI with their microscopic substrate in MS is not new [2]. However, the technology of both histology and MRI has significantly evolved including advances in co‐registration of the two modalities—MRI and histology—from mere visual like‐for‐like matching, through the use of a stereotaxic frame system [3] to current state‐of‐the‐art 3D printing technology using whole brain cutting boxes [4].

In this issue of Brain Pathology two international teams are taking us another step towards non‐invasively dissecting specific microstructural features in post mortem MS brain, ultimately serving the prospect that the severity of lesions, nature of inflammation and, in particular, the degree of (re‐) myelination can be inferred from quantitative MRI (qMRI) indices.

Galbusera and co‐workers focus on patterns of qMRI measures to (i) try and distinguish histological lesion types, and (ii) explore the relationship between those qMRI measures and quantitative histological indices of myelin, axons, and astrogliosis [5]. For this purpose, they employed a total of six different qMRI techniques including proton density‐weighted, quantitative T1 (qT1), magnetisation transfer ratio (MTR), myelin water fraction (MWF), susceptibility mapping (QSM), and diffusion‐derived metrics, such as fractional anisotropy (FA) and radial diffusivity (RD), on three whole post mortem brains from pwMS who had passed away with a relapsing (2) and secondary progressive (1) clinical phenotype, respectively. After scanning, regions of interest were defined on 3D echo‐planar MRI, which then guided tissue block dissection for immuno‐/histochemistry. Lesion classification included active, chronic‐active, inactive and remyelinated.

QSM, qT1 and—with some reservation due confounding by crossing fibres—RD came out on top distinguishing (i) active and (ii) remyelinated lesions versus the other lesion types. None of the MRI techniques employed was effectively separating inactive from chronic active lesions. However, both MTR and MWF were strong predictors of myelin in lesions whilst FA was modestly associated with axonal content, in both lesions (all types) and normal‐appearing white matter (NAWM). Alongside the presence of iron‐laden microglia/macrophages as the key substrate of paramagnetic rim lesions (PRL), Galbusera et al. confirmed the destructive nature of chronic active lesions compared to chronic lesions without rim, some of which displayed remyelination. Another interesting finding in their study was the association between MWF and astrocyte immunoreactivity, alongside extensive gliosis in remyelinated lesions. Evidence suggests a regulatory effect on microglial debris removal by astrocytes. Such removal is a likely precondition for successful remyelination.

The motivation for the study by Wiggermann and co‐workers is two‐fold. First, they are keen to validate the more recent 3D multi‐echo T2 gradient‐and spin‐echo (GraSE) technique over the traditional spin‐echo Carr–Purcell–Meiboom–Gill (CPMG) sequence for myelin water imaging (MWI), the main difference being that GraSE can be acquired in approximately half the time required for CPMG [6]. Whilst post mortem validation had been undertaken in the past for CPMG, this had not been the case for GraSE. For comparison with both MWI techniques, magnetisation transfer ratio (MTR), a widely used technique to quantify macromolecular water (and, by inference, the amount of macromolecules) was also acquired.

Second, and similar to Galbusera et al., Wiggermann are keen to classify MS lesions, and to detect various levels of myelin preservation and/or loss in lesions, diffusely abnormal and normal appearing white matter, using MRI [6]. Post‐mortem brain samples of seven pwMS, all with a progressive MS phenotype, were used. For registration of histology sections treated with different (immuno‐) stains, and histology‐MRI matching, 2D FMRIB's Linear Image Registration Tool (FLIRT) and NitfyReg were employed. Regions of interest (ROI) were identified on histology sections and then mapped onto MRI.

Strong association was detected between (i) myelin content measured using optical density on luxol‐fast blue stained sections and (ii) CPMG as well as GraSE suggesting both MWI techniques are useful substrates of myelin in the MS brain, with a time advantage of GraSE. That being said, the long‐established ‘workhorse’ of macromolecular quantification, MTR, performed rather well as a marker of myelin content too. Though confounders of MTR in the MS brain, including oedema and inflammation, appear to be more numerous than for MWI, the most important source of variability—axonal count—is, as Wiggermann and co‐workers rightly state, itself strongly associated with the amount of myelin. Thus, the use of MTR in clinical trials focussing on myelin repair still appears justified [7]. There is additional food for thought in both papers, for example, that breast carcinoma‐amplified sequence 1 (BCAS1) positivity appears to be not exclusive for remyelinating oligodendrocytes, but may also tag degenerating glial cells [6].

Both papers discussed here particularly highlight the need to further explore, in a larger sample, the nature and significance of PRL using QSM and other qMRI techniques given the current lead technology to detect PRL, positron emission tomography using the mitochondrial 18‐kDa translocator protein ligand [8], whilst being highly tissue and cell type‐specific, retains its well‐known issues in terms of resolution and multiple time point testing in vivo. PRL have become a truly ‘hot topic’ in MS research given slow expansion of at least some PRL may contribute to ongoing axonal injury thereby driving chronic disease deterioration [9]. For several of the main disease‐modifying drug makers in the field of MS, slowly expanding PRL and the activity state of microglia/macrophages have become a major focus in their argument underpinning the efficacy of Bruton tyrosine kinase (BTK) inhibitors in MS, a number of which are going to report phase III clinical trial data in the near future [10].

## AUTHOR CONTRIBUTIONS

This is an invited commentary, which has been generated and finalised by the author with no external contributions.

## CONFLICT OF INTEREST STATEMENT

I am the Chief Investigator of AttackMS (NCT05418010), which uses lesion magnetisation transfer ratio (MTR) as a co‐primary outcome. AttackMS is a multicentre clinical trial of natalizumab in people with clinically isolated syndrome suggestive of multiple sclerosis (MS), and people with a first manifestation of MS. The trial is sponsored by Queen Mary University of London and funded by Biogen Idec Limited, UK. I have received honoraria for presentations and advisory activities on BTK inhibitors from Merck and Sanofi.

## Data Availability

Data sharing is not applicable to this article as no new data were created or analyzed in this study.
